# Design of COSMIC: a randomized, multi-centre controlled trial comparing conservative or early surgical management of incomplete cervical cord syndrome without spinal instability

**DOI:** 10.1186/1471-2474-14-52

**Published:** 2013-01-31

**Authors:** Ronald HMA Bartels, Allard JF Hosman, Henk van de Meent, Jeannette Hofmeijer, Pieter E Vos, Willem Bart Slooff, F Cumhur Öner, Maarten H Coppes, Wilco C Peul, André LM Verbeek

**Affiliations:** 1 Department of Neurosurgery, Radboud University Nijmegen Medical Centre, R. Postlaan 4, 6500 HB, Nijmegen, The Netherlands; 2 Department of Orthopedics, Radboud University Nijmegen Medical Centre, Theodoor Craanenlaan 7, 6525 GH, Nijmegen, The Netherlands; 3 Department of Rehabilitation, Radboud University Nijmegen Medical Centre, R. Postlaan 4, 6500 HB, Nijmegen, The Netherlands; 4 Department of Neurology, Rijnstate Hospital, Wagnerlaan 55, 6815 AD, Arnhem, The Netherlands; 5 Department of Neurology, Radboud University Nijmegen Medical Centre, R. Postlaan 4, 6500 HB, Nijmegen, The Netherlands; 6 Department of Neurosurgery, University Medical Centre Utrecht, Heidelberglaan 100, 3584 CX, Utrecht, The Netherlands; 7 Department of Orthopaedics, University Medical Centre Utrecht, Heidelberglaan 100, 3584 CX, Utrecht, The Netherlands; 8 Department of Neurosurgery, University Medical Centre Groningen, Hanzeplein 1, 9700 RB, Groningen, The Netherlands; 9 Department of Neurosurgery, Leiden University Medical Center, Albinusdreef 2, 2333 ZA, Leiden, The Netherlands; 10 Department of Epidemiology, Biostatistics, and Health Technology Assessment, Radboud University Nijmegen Medical Centre, Geert Grooteplein 21, 6525 EZ, Nijmegen, The Netherlands

## Abstract

**Background:**

Incomplete cervical cord syndrome without spinal instability is a very devastating event for the patient and the family. It is estimated that up to 25% of all traumatic spinal cord lesions belong to this category. The treatment for this type of spinal cord lesion is still subject of discussion. From a biological point of view early surgery could prevent secondary damage due to ongoing compression of the already damaged spinal cord. Historically, however, conservative treatment was propagated with good clinical results. Proponents for early surgery as well those favoring conservative treatment are still in debate. The proposed trial will contribute to the discussion and hopefully also to a decrease in the variability of clinical practice.

**Methods/Design:**

A randomized controlled trial is designed to compare the clinical outcome of early surgical strategy versus a conservative approach. The primary outcome is clinical outcome according to mJOA. This also measured by ASIA score, DASH score and SCIM III score. Other endpoints are duration of the stay at a high care department (medium care, intensive care), duration of the stay at the hospital, complication rate, mortality rate, sort of rehabilitation, and quality of life. A sample size of 36 patients per group was calculated to reach a power of 95%. The data will be analyzed as intention-to-treat at regular intervals, but the end evaluation will take place at two years post-injury.

**Discussion:**

At the end of the study, clinical outcomes between treatments attitudes can be compared. Efficacy, but also efficiency can be determined. A goal of the study is to determine which treatment will result in the best quality of life for the patients. This study will certainly contribute to more uniformity of treatment offered to patients with a special sort of spinal cord injury.

**Trial Registration:**

Gov: NCT01367405

## Background

Traumatic Central Cord Syndrome (TCCS) was considered as a separate clinical entity. The most characteristic feature is disproportionately more motor impairment of the arms (especially the hands) than the legs, bladder dysfunction and varying degrees of sensory loss below the lesion.

Recently, it has been shown that the distinction of TCCS with an incomplete cervical spinal cord lesion (ICSCL) is artificial [1]. It is the most frequent incomplete traumatic spinal cord lesion. It accounts for up to 70% of all incomplete cervical spinal cord lesions [2], although the exact incidence is not known.

Uncertainty exists about the treatment. A good recovery has been described after conservative treatment [3]. Conservative therapy was usually considered when a fracture or dislocation of the spine was absent. It is often seen in hyperextension trauma in the elderly, with a degenerative spondylotic stenotic cervical spine. However, some reports suggest a better outcome after surgical decompression [4]. Randomized trials have not been performed.

Since the economic and emotional impact of a spinal cord lesion is enormous, optimal treatment should be instituted immediately after the traumatic event. To provide more evidence in the choice of a treatment for a specific traumatic spinal cord lesion, this study has been designed.

## Methods/Design

### Ethical approval

The study has been approved by the regional ethical board CMO Arnhem – Nijmegen.

### Goal of the study

This study will evaluate two strategies to improve functional outcome in patients with ICSCL without a fracture or instability of the cervical spine: 1) early decompressive surgery, and 2) conservative treatment.

### Eponym

COSMIC is an eponym for Conservative or Surgical treatment for Incomplete Cord lesion.

### Definition ICSCL without spinal instability

ICSCL is an incomplete spinal cord lesion due to a cervical spine trauma. Spinal instability is considered when the criteria defined by White and Panjabi are present [5]. At MRI and CT scanning with multiplanar reconstruction signs are not seen that could indicate a fracture of the cervical spine or instability. An overt sequestrated herniated disc should not be present since this will always necessitate immediate surgery. Involvement of the cervical spinal cord should be established at physical examination (symptomatic arm or hand dysfunction is obligatory).

### Surgical strategy

Several predictive factors for a good outcome have been identified. One is the timing of surgery after the injury. The debate about early versus late decompression is still not closed. Early has been defined rather broadly from less than 24 hours to less than 14 days [3,4,6].

From a biological point of view, it seems logical to decompress a neural structure as soon as possible to provide an optimal chance for recovery of the function. Since most patients will not be directly referred to the hospital where the surgical treatment will be offered, time for transfer of the patient should be included. Therefore in this study, early decompression is defined as within 24 hours after the injury. A recent meta-analysis on timing of decompressive procedures in cases of traumatic spinal cord injuries accompanying spinal fractures or dislocations, also considered a surgical decompression within 24 hours post-injury as early [7]. In this trial, patients allocated to the surgical strategy will be offered surgery within 24 hours post-injury. The main goal of the surgery is adequate decompression of the spinal cord.

### Hypotheses

H_0_: In patients with ICSCL without spinal instability, functional outcome for both strategies is the same.

H_1_: In patients with ICSCL without spinal instability, the early surgical strategy improves functional outcome compared to conservative treatment.

### Patient selection

To avoid discussion about possible confounding or effect modification related to the mechanism of trauma, this study will focus on ICSCL in patients without fracture or instability of the cervical spine on radiological examinations. Instability is established with MRI, CT, lateral and anteroposterior plain radiographs of the spine. In case of doubt, radiographs in sitting position or dynamic radiographs are made under direct supervision of the treating surgeon.

Inclusion criteria: all adult patients with a history of a traumatic event to the cervical spine fulfilling the criteria of ICSCL.

Exclusion criteria: cognitive impairments, known neurological disease, psychiatric illness, significant co-morbidity interfering with the indication to perform surgery or not, use of anti-coagulating drugs, addiction to drugs or alcohol (more than five units daily), not speaking Dutch language fluently, not willing to participate or participating in another trial.

### Treatment allocation

After written informed consent has been obtained patients will be randomized to either surgical or conservative treatment. Informed consent should be obtained within two hours after arrival at the emergency department. Although no mode of treatment has been proven superior to the other, surgical decompression may be warranted during the conservative strategy treatment. It is not considered as a failure but as a part of the conservative strategy.

### Interventions

Surgical treatment will consist of decompression of the spinal cord by an anterior, posterior or combined approach. The choice for the approach is made by the treating surgeon. All participating surgeons are experienced in spinal surgery. Fixation may be added to the decompressive surgery according to the preference of the treating surgeon. Procedure and use of implants will be documented. If a patient is allocated to the surgery group it should be executed within 24 hours post-injury. Postoperatively, an intensive rehabilitation program will be prescribed. This will include exactly the same elements as for those patients who were allocated to the conservative treatment.

Conservative treatment will consist of an intensive rehabilitation program including physiotherapy and ergotherapy. However, when a patient deteriorates after 24 hours post-injury, surgery may be indicated. This decision is made by the treating physician. Should a patient be operated upon after he has been located to the conservative group, it is not considered as a failure of the conservative strategy, but as a possible consequence in case of deterioration.

### Outcomes

Primary outcome: functional outcome at two years as measured by validated Dutch translation of mJOA.

Secondary outcomes: motor and sensory scores according to the ASIA standards at six weeks, 12 weeks, 12 months, duration of the stay at a high care department (medium care, intensive care), duration of the stay at the hospital, complication rate, mortality rate, sort of rehabilitation, quality of life, SCIM III, and arm/hand function assessed by the disability of the arm, shoulder and hand questionnaire (DASH).

Furthermore, subjects keep a diary on the financial consequences and health care consumption related to ICSCL. Visits to the general practitioner, physical therapist, medical specialists, alternative health practice, use of analgesics, duration of sick leave from work and additional utility/mobility costs are recorded. The patient’s or diaries are returned during the outpatient clinic visits or by mail.

### Scales

#### mJOA

The scale was developed for evaluation of impairments related to degenerative spondylotic changes in the cervical spine. Nevertheless, this scale is very useful since it suitably describes the functional limitations of a patient with compromised function of the cervical spinal cord. The translation into the Dutch language has been validated [8]. The minimal clinical important difference has been estimated [9]. Since the patient can complete the questionnaire himself, the scale is functional and a minimal clinical important difference has been estimated, the mJOA is used as a primary outcome measurement.

#### SCIM III

The Spinal Cord Independence Measure (SCIM III) is also a functional score that has been developed to evaluate the functional recovery of patients with a spinal cord lesion. It can be easily completed even by phone. Recently, its reliability and validity has been shown [10] however, a minimal clinical important difference has not been established.

#### ASIA

The score of the American Spinal Injury association (ASIA score) is the most frequently used scale to grade neurological level and the severity of spinal cord injury. The ASIA motor score represents the muscle strength of determined muscles in arms and legs and the ASIA sensory score represents the light touch and pin-prick sensory functions within all dermatomes. The score is reduced to upper extremity motor score (range 0–50), lower extremity motor score (range 0–50) and sensory score (range 0–112).

#### EuroQol 5QD (EQ-5D)

EQ-5D is a standardized health-related quality of life questionnaire that can be applied to a wide range of health conditions and treatments. It is designed for self-completion by respondents. It consists of a descriptive part compromising five dimensions: mobility, self-care, usual activities, pain/discomfort and anxiety/depression, and a VAS score indicating the self- rated health on a 20 cm VAS scale.

#### DASH

The DASH was developed as a 30-item questionnaire to be completed by the patient to assess functional status for clinical use in daily practice or as a research tool. DASH evaluates symptoms and physical function with a five-response option for each item. It has been proven to be a reliable and valid instrument to assess disability in patients with a variety of upper limb disorders [11]. The Dutch translation has been validated [12].

### Evaluation patients

The nature of the treatment under in the study prevents a completely blind evaluation of the outcome. Therefore, an independent observer trained in neurological examinations, will perform the physical examination at each follow- up visit. He/she will also complete the questionnaires and check the self-assessed questionnaires on completeness (Table 1). The independent observer will not participate in the initial admission, randomization and treatment of the patient.

**Table 1:** Study measurements over time

	**Admission**	**6 weeks post**-**injury**	**12 weeks post**-**injury**	**12 months post**-**injury**	**24 months post**-**injury**
Patient demographics	X				
ASIA	X	X		X	X
mJOA	X	X	X	X	X
SCIM III	X	X	X	X	X
DASH	X	X		X	X
EQ-5D	X	X	X	X	X
Patient diary	X	X	X	X	X
Complication registration		X	X	X	X
MRI	X				
CT with reconstruction	X				

### Data collection

Data will be collected during/each patient contact through an internet based database. Investigators have access to data, but do not have the possibility to check the results during the trial. Neither do they have any possibility to correct data. If required, data can be changed by an independent supervisor of the database.

### Design

The study has been designed as a multi-centre, open, randomized controlled trial. Participating centres should have adequate experience with the treatment of patients with spinal cord trauma and neurosurgical facilities should be available on a 24 hours-a-day basis. All primary analyses will be performed on an intention-to-treat basis comparing the two strategies.

### Randomization

To improve the balance in the number of treatment assignments in each participating centre, randomization will be performed in blocks. A computer generated list of treatment allocation is used for randomization. Treatment assignment is made by secure computer access.

### Sample size

Power calculations are hampered by the scarcity of data on clinical outcome in this patient group. The minimal clinically important difference in mJOA has been estimated in an earlier trial [9,13]. A difference of at least two points on the modified JOA functional score is considered significant. The difference is expected to be mainly allocated to the function of the arms and legs. Based on literature, a standard deviation of 2 is assumed. This estimate was confirmed by asking three experienced cervical spine surgeons (members of the board of the CSRS-es).

The sample size is calculated as follows: a two group student test with a 0.05 two sided significance level and a power of 95 % will need a sample size per group of 27 to detect a significant difference. Considering a ten percent loss in follow up a total of 30 individuals per group need to be included.

During the conservative treatment some patients will require surgical decompression due to for example neurological deterioration. It is estimated that it will occur in 20% of the patients. Although this is not considered a failure of treatment, the sample is increased to 36 patients per group in order to maintain the same power.

Only patients allocated to the conservative group who are offered surgery within 24 hours post-trauma, are considered crossovers. This number is expected to be very low. However, after completion of the trial this will be evaluated and a separate per-protocol analysis performed if significant cross over occurred.

Five centres in the Netherlands will participate and is estimated that its conclusion will be within 2 years.

### Statistical analysis

Baseline characteristics are reported using descriptive statistics. Any imbalance characteristics found visually will be account for in analysis of primary outcome.

Since the primary outcome of the two strategies is compared after two years, a test for independent samples will be used (student-t test). To see if differences at all time points are present, these are assessed by computing both the main effect of the treatment and the interaction between treatment and time in a mixed linear model.

## Discussion

Although already described in 1887 [14], traumatic central cord lesion has first been defined by Schneider in 1954 [15]. It was characterized by disproportionately more motor deficit of the arms and especially the hands than the legs, bladder dysfunction and varying degrees of sensory loss below the lesion. It was most frequently seen in hyperextension injuries causing squeezing or pinching of the spinal cord both anteriorly and posteriorly. Since its definition it was considered as a separate entity. Recently, it was shown that it is just a form of incomplete spinal cord lesion [1] therefore we abandoned the use of the term traumatic central cord syndrome.

Since the description of traumatic central cord syndrome its treatment has been the subject of discussion. In 1954 surgical decompression was even contraindicated because spontaneous improvement or complete recovery may occur [15]. At this moment either surgical decompression or conservative treatment are favoured. If a surgical decompression is chosen, the timing of the procedure is also debated.

From a biological standpoint early decompression seemed to be the most effective due to the prevention of secondary damage [16]. In this study early surgery is defined as decompression within 24 hours. Due to referral patterns it was thought that a more strict time schedule would negatively interfere with the inclusion of the patients. The operative approach to decompress will be dependent on the surgeon. It is thought that the approach does not contribute to the clinical effect as long as the spinal cord is decompressed effectively.

In this study, surgical decompression is solely compared to a standardized conservative treatment. The conservative treatment offered in both treatment arms will be equal.

As primary endpoint the score at the translated mJOA was chosen. Although it was developed for evaluation of symptomatic cervical myelopathy due to degenerative changes, its simplicity and the evaluation of the functional impairment by the mJOA justified the choice above the others. The scale is completed with the help of the patient or even by the patient self. The mJOA addresses the function of the upper limbs, lower limbs and urinary sphincter. In fact, the signs and symptoms of cervical spondylotic myelopathy resemble those of an incomplete spinal cord lesion. Furthermore, functional outcome is the most important for the patient treated for a spinal cord lesion as it will directly influence his/her quality of life.

The ASIA score has been designed for the evaluation of traumatic spinal cord lesion, however it was not a functional score. On the other hand, SCIM III has recently been developed to evaluate the functional impairment [10]. However, the experience with the scale is limited and a clinically minimal important difference has not been established. It will be measured at each visit and its value in comparison to the other scales, including the scales for quality of life, will be established.

Since the function of the hand is generally more affected than the function of the lower limbs in ICSCL, extra attention is given to a more specific test for studying the function of the hand. For this purpose, a questionnaire to be self assessed was chosen, DASH. Apart from the self assessment the validation of the translation into Dutch is an advantage.

The major goal of the study will be to compare clinical outcome measured by functional scales and quality of life scales between two established treatment options. Parallel to this objective, direct and indirect costs will be evaluated from a societal perspective. Analysis of clinical outcome in relation to costs is part of the study.

Several problems may arise. The recruitment of patients may be hampered by the emotional distress during the time that the patient must be included. In addition to an informed consent form a legal representative is also required, but this does not preclude a delay in the inclusion since the legal representative may be in emotional distress as well. Furthermore, MRI may disclose findings like hemorrhage within ligaments or discs or other abnormalities suggesting instability. Even if instability is not shown on the standard radiographs, surgeons might be willing to offer the patient surgery. Although a patient could have been included in the trial, surgeons’ preference prohibited inclusion. Participating surgeons are encouraged to follow the protocol. In case of doubt, the project leader is always willing to deliberate the case including the findings at radiographs. However, it cannot be prevented that potential candidates are sometimes not included due to a surgeon’s preference. The freedom to choose for a surgical approach including the use of implants may interfere with the results. However, since every patient is unique a standard approach cannot be given. Therefore, a more pragmatic approach is chosen requiring adequate decompression of the spinal cord.

## Competing interests

The authors declare that they have no competing interests.

## Authors’ contributions

RHMAB contributed to conception, design, drafting the manuscript, and final version, AJFH, HM, and JH contributed to conception, design and revising the draft, PEV, WBS, FCO, WCP contributed to revising the draft and finalizing the manuscript, ALMV participated in the design of the study. All approved the final version. At this moment funding of the study has not been established yet.

## Pre-publication history

The pre-publication history for this paper can be accessed here:

http://www.biomedcentral.com/1471-2474/14/52/prepub
